# A Manual of Diseases of the Ear

**Published:** 1889-07

**Authors:** 


**A Manual of Diseases of the Ear,** for the Use of Students and Practitioners of Medicine, by ALBERT H. BUCK, M. D., Clinical Professor of the Diseases of the Ear, in the College of Physicians and Surgeons, New York; Consulting Aural Surgeon New York Eye and Ear Infirmary. 420 pages. Illustrated. Price, extra muslin, $2.50. New York: William Wood & Co.

Dr. Buck tells us that since the publication in 1880 of his work entitled "Diagnosis and Treatment of Ear Diseases," he has been led by further experience to modify in some important respects the views therein expressed and the methods of treatment therein advocated. The present volume contains, therefore, his latest opinions on the important subject of the Diseases of the Ear, and has been so arranged to meet the wants of medical students as well as practitioners of medicine. It appears as the first of a series of American Text-Books announced by the well-known publishing house of William Wood & Co., of New York. We believe it will be welcomed both by teachers and students, as its author is a careful observer and a physician of large experience in this special department. As a whole, the book deserves unqualified praise. Throughout its pages runs a wise conservatism. The author is neither timid nor rash—neither tied to old opinions nor rushing heedlessly after every new one. In a word, he is a safe teacher.

Without presenting an analysis of each chapter, we desire to call attention to some of the statements contained in a few of them. The chapter on General Pathology is most instructive. Its generalizations are eminently sound, as witness the following: "Putting to one side, for the moment, the few other diseases or abnormal changes which may take place in the ear, we may say that it is the chief task of aural pathology to set forth and make plain the different changes which inflammation may produce in the three different cavities described above. We shall get a much clearer idea of 'diseases of the ear' if we grasp and hold on firmly to this simple conception of a single morbid process, which, however, may vary greatly in its essential character, in its localization, and in its issues." And again, in the chapter on Diseases of the Middle Ear he says: "The great majority of diseases of this part of the ear are essentially inflammatory in character, and with scarcely any exception they originate in an inflammation of the vault of the pharynx." Nothing is more important for the student to appreciate than the part inflammation plays in the diseases of the ear, and Dr. Buck rightly takes every opportunity to fix the attention on that feature of aural pathology.

In the description of the methods of examination, and in the value to be placed on the various diagnostic tests, the author is generally clear and sound. We are, however, somewhat astonished at the statement (p. 143) that the author seldom uses posterior rhinoscopy as an aid to diagnosis in diseases of the ear. This is all the more remarkable as he recognizes the importance of knowing the condition of the naso-pharynx, and certainly no other method of examination will reveal that condition so easily. Again, the condition of the pharyngeal end of the Eustachian tube is best ascertained by posterior rhinoscopy; it is safer and easier to apply than any other method we know. We cannot agree with Dr. Buck that Zaufal's specula have further perfected the method of direct examination. They are not of much practical value, as their introduction presupposes a nasal cavity straight as to its bony parts, and this condition is not often seen in those cases where an examination of the naso-pharynx is most important. Their use certainly cannot be compared with the method which Dr. Buck says he seldom uses as an aid to diagnosis in diseases of the ear.

Some of the most important chapters relate to treatment. A large number of illustrative cases have been introduced, and they add much to the value of the book. In some instances the treatment in these reported cases differs from that advocated in the text, and where this is so, Dr. Buck says the student must follow the treatment advised in the body of the work. This is very fine, and is another example of the care the author takes to give the student the benefit of his large experience.

Many other parts of the work could be praised in equal terms. We are convinced that Dr. Buck has given the profession, and especially the teaching part of it, a book which will grow in favor the more it is used, and which will long remain a standard authority. The volume is well bound and printed—the reports of cases being in smaller type than the general text. The illustrations are very good, and many of them are new. A satisfactory index completes the work. We await with interest the other volumes of the series, and expect they will maintain the high degree of excellence of this first one.

